# Extremely High-Throughput Parallel Microfluidic Vortex-Actuated Cell Sorting

**DOI:** 10.3390/mi12040389

**Published:** 2021-04-02

**Authors:** Alex A. Zhukov, Robyn H. Pritchard, Mick J. Withers, Tony Hailes, Richard D. Gold, Calum Hayes, Mette F. la Cour, Fred Hussein, Salman Samson Rogers

**Affiliations:** 1 Cellular Highways Ltd., Melbourn Science Park, Melbourn, Cambridgeshire SG8 6EE, UK; alex.zhukov@cellularhighways.com; 2 TTP PLC, Melbourn Science Park, Melbourn, Cambridgeshire SG8 6EE, UK; robyn.pritchard@cellularhighways.com (R.H.P.); mick.withers@ttp.com (M.J.W.); tony.hailes@ttp.com (T.H.); richard.gold@ttp.com (R.D.G.); calum.hayes@ttp.com (C.H.); mette.lacour@ttp.com (M.F.l.C.); fred.hussein@ttp.com (F.H.)

**Keywords:** microfluidics, cell sorting, high-throughput, VACS, cytometry

## Abstract

We demonstrate extremely high-throughput microfluidic cell sorting by making a parallel version of the vortex-actuated cell sorter (VACS). The set-up includes a parallel microfluidic sorter chip and parallel cytometry instrumentation: optics, electronics and control software. The result is capable of sorting lymphocyte-sized particles at 16 times the rate of our single-stream VACS devices, and approximately 10 times the rate of commercial cell sorters for an equivalent procedure. We believe this opens the potential to scale cell sorting for applications requiring the processing of much greater cell numbers than currently possible with conventional cell sorting.

## 1. Introduction

The speed or throughput of conventional fluorescence-activated cell sorting has been a severe limitation for important applications, including cell therapy, liquid biopsy and high-throughput phenotypic screening. For example, cell therapies, such as autologous chimeric antigen receptor (CAR) T-cell therapies, often demand the selection of a rare phenotype at >90% purity from an input population exceeding 10^9^ cells, to produce efficacious doses of ~10^9^–10^10^ cells [1,2,3]. Besides speed, other important improvements over conventional cell sorters, such as automation, reproducibility and sterility, are required for clinical applications.

Conventional cell sorting originated from continuous inkjet printing technology, to sort particles by the electrostatic deflection of droplets created by the stimulation of the Plateau–Rayleigh instability of a pressurised stream ejected from a nozzle and into the atmosphere [4]. Since the only common short name for this technology is the acronym “FACS” (fluorescence activated cell sorting), which is a trademark of Beckton, Dickenson and Company, we refer to the conventional technology instead by the acronym CICS (continuous inkjet cell sorting), which reflects the physical principle of droplet deflection and the origin of the technology, rather than the “fluorescence activation”, which is shared by many microfluidic cell sorters that use quite different mechanisms for particle deflection [5,6,7,8,9,10,11,12,13,14,15,16,17]. The throughput of CICS is not limited by the rate at which droplets can be produced, but by the viability of cells [18,19]. For lymphocytes and typical small mammalian cells, viability drops above rates of 30,000–70,000 droplets/s, using 100–70 µm nozzles respectively. To achieve a high yield in purity mode sorting, users feed the sorter with a lower rate of cells than the droplet rate, to ensure that there is a high proportion of droplets containing only one cell. Typically up to 1/3–1/4 of the droplet rate is used, i.e., 10,000–20,000 cells/s. Microfluidic cell sorters based on various deflection mechanisms have been published, including those using acoustic forces [5,6,7,8], magnetically actuated mechanical valves [14], dieletrophoretic forces [15,16,17], and liquid deflection with thermal vapour bubbles or other transducers [10,11,12,13,20].

There is good reason to believe that any flow-based single-stream sorter will cause a reduction in cell viability at similar rates, since the shear or extensional flow rates that cause mechanical stress to a cell are also directly related to the deflection of packets of liquid containing individual cells [18,19]. Therefore, the main paradigm for achieving much faster cell sorting is the parallelization of streams. It is a great practical challenge to achieve parallel sorting, since it also requires the parallelization of cytometry instrumentation across all channels. For example, ~0.5 × 10^9^ cells/hr would be satisfied by 16 sorters, each processing 10,000/s. However, arranged as a 4 × 4 array on chip, the pitch must be ≤1 mm to achieve sufficient sensitivity with reasonable optical components (lenses and filters ≤2-inch diameter). While several parallel flow cytometers [21,22,23,24,25,26,27] and one parallel sorter [20] have been published, no previous microfluidic cell sorters met this specification because of their size (including actuators and side-channels), low sort rates, or other complexities.

Vortex-actuated cell sorting (VACS) was described in our previous article [28]. This cell sorting technology employs a thermal inkjet-style microheater, which is attractive as a transducer of electrical to mechanical energy within a microfluidic chip due to its high power, small size, and maturity of associated fabrication processes. However, the key innovation of our previous article was to couple this transducer to generation of a transient vortex within the flow channel to deflect individual cells. What distinguishes VACS from the other microfluidic technologies that use standing vortices for cell separation [29,30,31,32,33] is the transience of the vortex in VACS, which enables the sorting of individual particles within the flow. (Note that previous microfluidic devices based on standing vortices are not cell sorters in the sense of “fluorescence activated cell sorting”.) We also used serpentine inertial focusing in the input channel to focus cells to the centre of the stream [34]. The result was a microfluidic cell sorter of speeds comparable to CICS with high viability cell output: i.e., 23 µs sort envelope (equivalent to droplet rate of 1/(23 µs) = 43 kHz), with a chip footprint of approximately 1 mm × 0.25 mm, including the actuator but excluding the inertial focuser. We included a preliminary design of parallel cell sorter chips based on VACS.

The purpose of this paper is to report the first working parallel VACS chip, consisting of 16 individual VACS devices operating asynchronously, showing also how the cytometry instrumentation can be parallelized to work with such a chip. The result is extremely fast, achieving approximately ten times the typical throughput of current commercial cell sorters of an equivalent procedure, representative of a high rate sorting of lymphocytes or small mammalian cells.

## 2. Materials and Methods

**Figure 1:**
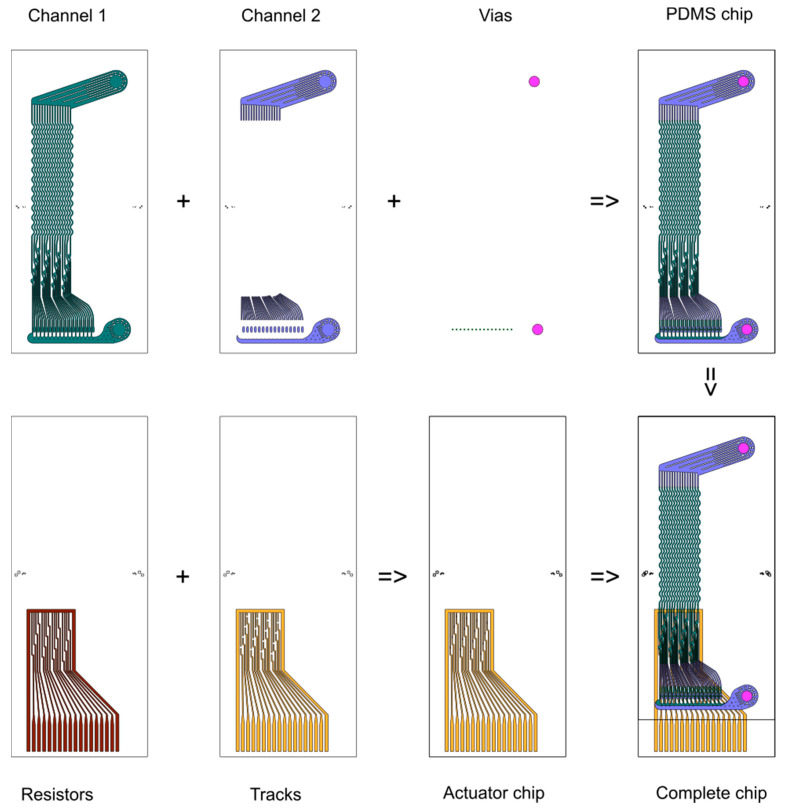
Design of the parallel vortex-actuated cell sorter (VACS chip). Top left to right: a microchannel layer was cast in PDMS using a two-layer mould (Channel 1–2 layers); vias were cut in the PDMS using a biopsy tool (inlet and positive outlet) and laser ablation machine (negative outlets). Bottom left to right: a first sputtered layer of titanium on a glass substrate served as both the resistor material and an adhesion layer for the second layer of tracks. The complete chip was produced by alignment and bonding of the PDMS and glass chips.

**Figure 2:**
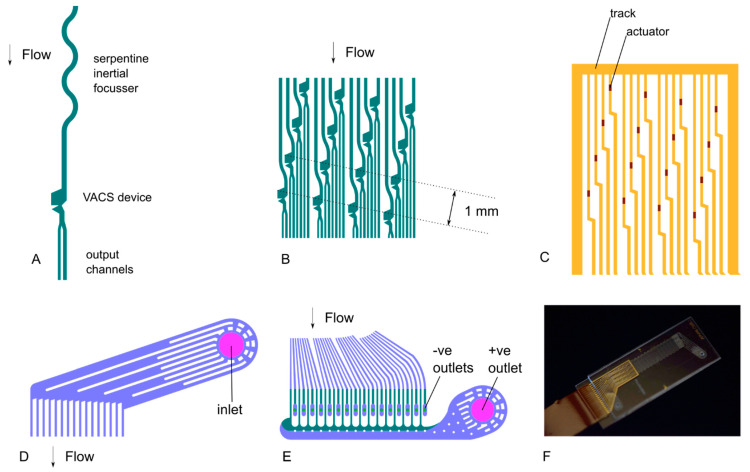
Important details of the chip design are shown. (**A**): individual sorter comprising serpentine inertial focuser, VACS device and output channels. (**B**): 4 × 4 array of VACS devices. (**C**): 4 × 4 array of microresistor actuators. (**D**): inlet manifold. (**E**): outlet manifolds. (**F**): photograph of the complete chip.

The design of the parallel VACS chip is shown in Figure 1. The chip consists of channels and vias in a cast polydimethylsiloxane (PDMS) slab and resistors, tracks and contacts made by sputtering onto a glass substrate. Full microfabrication methods were described in our previous article [28]. The PDMS slab and glass substrate, which are each 0.5 mm thick, are bonded together using oxygen plasma. Figure 2 shows important details of the chip: (A) the individual devices, (B) the array of sorter channels, (C) matching array of actuators, and the (D) input and (E) output manifolds. Figure 2F shows a photograph of the complete chip, also showing the flexible electronics connector bonded onto the gold contacts on the glass substrate.

Several technical problems were resolved: firstly all 16 sorter inputs branch from the same inlet, achieving a uniform split of flow and particles as described below. Secondly, the serpentine inertial focusers on the inlet channels stack efficiently together with an array pitch of 0.25 mm, while the VACS devices themselves are arranged on a 4 × 4 array of 1 mm pitch. Thirdly, we were able to substantially equalise flow resistance through each parallel sorter, by allowing the same length and cross section of each within a high-flow-resistance low-height channel (Channel 1 layer, 37 µm), while the input and output manifolds were made with a low-flow-resistance high-height channel (Channel 2 layer, 250 µm). Fourthly, we solved the topological problem of recombining the interleaved 16 positive and 16 negative output channels by merging only the positive channels within the chip. The negative output channels were designed so that they terminated in a high-height channel; these were then opened in the via layer using laser ablation. The 16 negative output streams then merged outside the chip in a specially-designed elastomeric compression fitting within a machined aluminium mount.

### 2.1. Microfabrication Processes

Channels were made in PDMS (Sylgard 184 silicone elastomer, Dow Corning Corporation, Midland, TX, USA) by casting from an SU-8 (SU-8 3035, Microchem) mould on a silicon wafer. In the present device, two layers of SU-8 were sequentially spin-coated (EMS 6000, Electronic Micro Systems Ltd., Salisbury, UK), patterned by photolithography (EVG 620, EV Group, St. Florian am Inn, Austria) and developed on the same substrate, to form a two-layer channel mould (Channel 1 and Channel 2 layers). The mould was exposed to PFOTCS ((triedecafluoro-1,1,2,2,-tetrahydrooctyl)silane, Merck Life Science UK Ltd., Glasgow, UK) to aid release of the PDMS from the mould. A measured quantity of PDMS was poured over the mould to cast a 0.5 mm slab thickness, then the liquid PDMS was vacuum degassed to remove all microbubbles from the PDMS. The mould was then placed on a level surface to allow a uniform casting thickness. The PDMS was cured at room temperature for 48 h: we avoided curing at higher temperatures to avoid any shrinkage when returning to room temperature.

Two electronic layers were made by sputter deposition (S060M, Moorfield Nanotechnology Ltd., Knutsford, UK) onto a Borofloat BF33 glass wafer (Schott AG, Mainz, Germany). Firstly, 200 nm titanium was deposited as the resistor layer, also fulfilling the role of an adhesion layer on the glass. Secondly, 500 nm gold was deposited as the track layer on top of the titanium. The layers were patterned by photolithography and wet-etching. Finally, the glass wafer was diced into individual chips.

The PDMS slab was then bonded to the glass substrate by oxygen plasma (RIE80-GA, JLS Designs Ltd., Somerset, UK), followed by manual alignment under an optical microscope in a specially designed four-axis micrometre stage jig (XYθZ). The output vias were subsequently opened by ablation with an excimer laser machining system (Optec Lightbench from Optec S.A., Frameries, Belgium).

### 2.2. Parallel Optical Cytometry

Parallel optical cytometry was made practical by the relatively small field-of-view of the 4 × 4 array of sorters, which fits in a 4 mm diameter. This enabled us to use a commercially available objective lens for imaging and fluorescence light collection from all devices on the chip simultaneously, as well as standard 1-inch lens tubes and filters.

**Figure 3:**
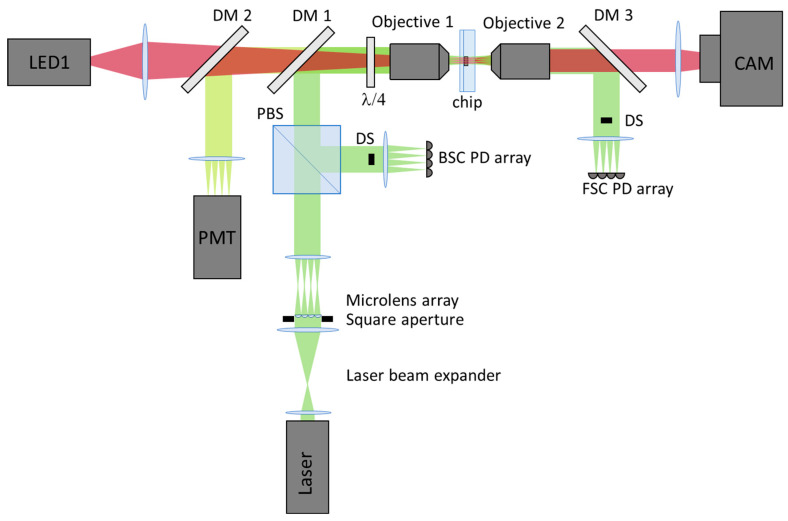
Architecture of the cytometry optics, including the excitation laser, the matched objective lenses, the chip, the array detectors for fluorescence (PMT) and scatter (BSC and FSC), and the strobe LED and camera.

A schematic of the optical set-up is shown in Figure 3. The overall architecture is very similar to the single-stream cytometry of our previous article [28], except that the excitation is now an array of laser spots and the detectors are arrays instead of single detectors.

Naming the optical axis as *Z*, the chip is mounted on a three-axis XYθ micrometre stage between two matched objective lenses (4X Super Apochromatic Microscope Objective, 0.2 numerical aperture, 17.0 mm working distance; TL4X-SAP from Thorlabs Inc., Newton, NJ, USA). Objective 1 delivers the excitation light to the chip and simultaneously collects fluorescence light to a PMT (photomultiplier tube) array and backscattered light (BSC) to a photodiode array. It also delivers light from a strobe light-emitting diode LED1 (800 mW emission centred at 940 nm; M940L3 from Thorlabs Inc.) to the chip. Objective 2 collects forward-scattered light (FSC) to a photodiode array and LED1′s light to a camera (acA2040-90 um from Basler AG, Ahrensburg, Germany). Dichroic mirrors DM1 and DM3 are long pass at 560 nm; FF560-Di01-25 × 36 from Semrock, (Idex Health and Science LLC, New York, NY, USA) are used to separate the excitation, FSC and BSC from the fluorescence. Dichroic mirror DM2 is used to separate the strobe imaging (long pass at 801 nm; FF801-Di02-25 × 36 also from Semrock), while tube lenses are used to focus the chip onto the PMT and photodiode array detectors and camera. The excitation light comes from a continuous wave laser 532 nm, producing up to 6 W output (Opus 532, Laser Quantum, Stockport, UK) and is expanded in two axes into an ellipse then split into an 4 × 4 square array of spots using a microlens array (Microlens Array Nr. 18-00672 from SUSS MicroOptics AG, Neuchatel, Switzerland). BSC is separated from the excitation laser using a quarter-wave plate (WPQ10M-532 is from Thorlabs Inc.) and polarizing beam splitter (CCM1-PBS251/M from Thorlabs Inc.). A specially-designed direct-light beam stop (DS) is placed in the infinity plane before each of the FSC and BSC detectors to remove the unscattered direct light and specularly back-reflected light, respectively.

We used a commercially available multianode PMT as the array detector for fluorescence (H12428 8 × 8 Multianode Photomultiplier Tube Assembly from Hamamatsu Photonics K.K., Japan), but made our own photodiode array for FSC and BSC, putting 16 photodiodes (S12158 Si PIN photodiodes, Hamamatsu Photonics K.K.) on a printed circuit board (PCB). Array detectors were mounted in an anodised metal case with an aperture plate in front of each detector to reduce stray light and facilitate easier alignment. Each detector box was mounted on an XYZ micrometre stage to allow focusing and alignment with respect to the image of laser spots. Since the multianode PMT was of double the required resolution (8 × 8), we used every second pixel row and column to reduce cross-talk between the pixels.

**Figure 4:**
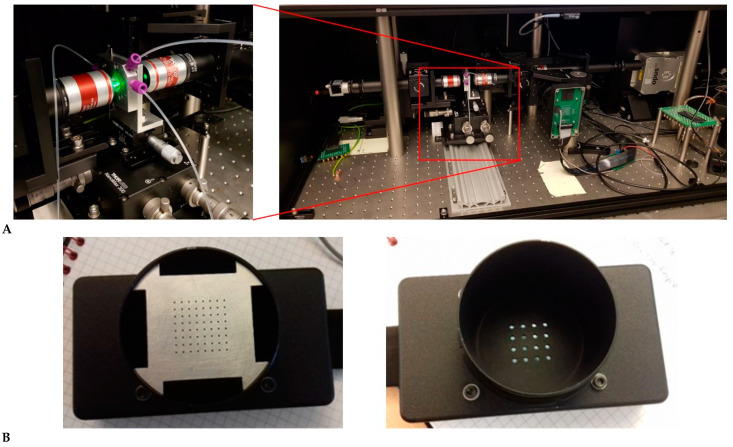
Photograph of the optics set-up (**A**) including a close-up of the mounted chip between matched objective lenses. Photographs of the array detectors (**B**) showing the mounted detectors behind an aperture plate.

Figure 4 shows (A) images of the whole optical set-up, a close-up of the chip mounted between the objective lenses, and (B) the detector boxes.

### 2.3. Parallel Control Electronics and Software

The control electronics was organized as a set of separate single-stream processing boards to process the data for each individual sorter. This set was mounted on a host board to route communication between single-stream boards, control outputs, and control personal computer (PC). Each single-stream processing board has a field programmable gate array (FPGA; Altera Cyclone IV EP4CE22F17C6N on a Terasic Cyclone IV Deo-Nano development module, Altera, San Jose, CA, USA) for real-time data processing and three 14 bit 8 MHz analog-to-digital converters (ADCs; THS1408 from Texas Instruments, Dallas, TX, USA) for input of FSC, BSC and fluorescence from the relevant pixel of the array detectors. The host board has a single FPGA (Altera Cyclone IV EP4CE75F23C8N) providing distribution of the input signals to the single-stream boards, and collection of the single-stream board outputs. These outputs were event data fed to the control PC via UDP over ethernet, and real-time control outputs (actuation trigger, strobe trigger, camera trigger) to the array of actuators, the strobe LED and camera.

**Figure 5:**
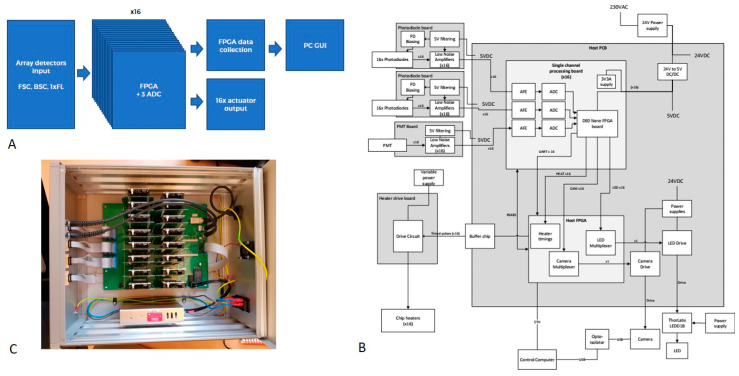
Schematic of the electronics architecture (**A**), a diagram of connectivity (**B**) and a photograph of the set-up (**C**).

Figure 5 contains a schematic of the electronics architecture (A), a diagram of connectivity (B) and a photograph of the set-up (C).

**Figure 6:**
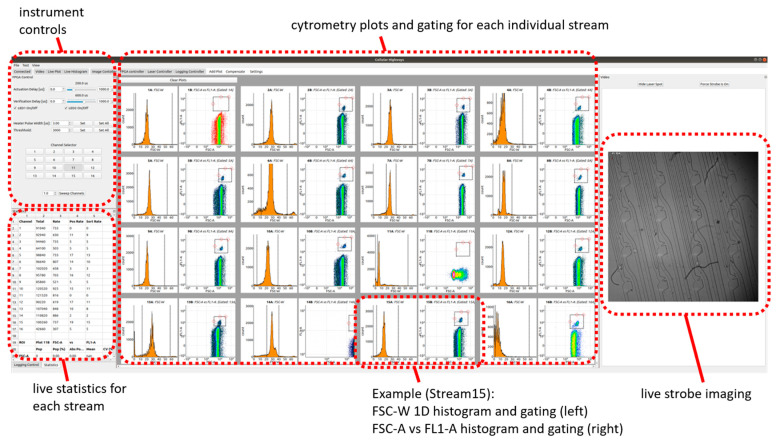
PC GUI allowing simultaneous display of event data, and cytometry gating from all parallel channels, via 1D and 2D histograms applied individually to each stream. This screenshot shows an instance of beads flowing through the device.

Figure 6 shows a PC graphical user interface (GUI) which we set up in Python to receive the event data from the host FPGA and provide cytometry gating and set-up parameters to the host FPGA. The camera data was fed to the PC directly via a universal serial bus (USB) connection. Each individual sorter is plotted, gated and manipulated independently.

Further details of the control system provided in our previous article are also representative of the present parallel system [28].

### 2.4. Testing the Sorter with Beads and Cells

To test the sorter, we connected a syringe pump (neMESYS 290N, CETONI GmbH, Korbussen, Germany) with a 20 mL syringe to the inlet, and collected the outputs in 50 mL centrifuge tubes. The positive output was connected to a solenoid pinch valve as an “unclogger mechanism”: when debris appears in the junction between the positive and negative output channels, transiently closing the positive output, using the solenoid valve sweeps this debris to the negative output.

Beads tested were 10 µm unlabelled and 10 µm green fluorescent monodisperse polystyrene particles (PS-R-10.0 and PS-FluoGreen-10.0, respectively, from Microparticles GmbH (Berlin, Germany). Green fluorescent beads were mixed with unlabelled beads to obtain suspensions that were 1–5% fluorescence positive.

Cells tested were HEK293T cells (unlabelled and green fluorescent protein (GFP) positive) (Merck Life Science UK Ltd.), which were cultured using Dulbecco’s Modified Eagle’s Medium (DMEM) supplemented with 2 mM Glutamine and 10% Fetal Bovine Serum (FBS), trypsinised and resuspended in Hank’s Buffered Salt Solution (HBSS, Merck Life Science UK Ltd.) for sorting. GFP positive cells were mixed with unlabelled cells to obtain suspensions of cells that were 1–5% positive. Cells were filtered with a 30 µm sieve for aggregates before sorting (pluriSelect Life Science UG (haftungsb.) & Co. KG, Leipzig, Germany).

Suspensions of cells and beads were adjusted to a concentration of 1–3 × 10^6^/mL for sorting.

## 3. Results

The apparatus consisting of the parallel VACS chip, sealed and mounted in the optical cytometry rig, with electronics, control PC and GUI attached, was sufficient to acquire initial results to confirm the raw potential of extremely high-throughput parallel cell sorting based on VACS.

While a bead or cell suspension was flowing, the operator would apply the gates of the FSC, BSC and fluorescence to each stream in turn, then switch on sorting. Using the strobe LED and camera to sample the sort events, the actuation pulse amplitude and duration were adjusted until the well-timed positive particle could be seen in the positive output channel, while all other particles streamed to the negative output channel. A double strobe was used to reveal the particle position, firstly at the moment of actuation, and secondly to verify deflection after passing the sorter junction, as previously described [28]. This procedure was repeated for each of the individual sorters. When all 16 individual sorters had been set up, the host FPGA was set to rotate the strobe LED and camera sequentially between each sorter, sampling 10 events on each to verify its correct operation.

The basic functionality was verified for both beads and cells. In particular, we observed that the parallel VACS set-up mitigated several technical risks that concerned us previously, which may have prevented the independent asynchronous operation of the individual sorters. These include the possibility of pressure or flow transients affecting sorting in neighbouring channels, the uneven distribution of particles or flow among the individual streams, difficulty in the three-dimensional alignment of the laser foci (array and plan) to the chip, and crosstalk in the cytometry channels (FSC, BSC, fluorescence) between the adjacent streams. The operation of the unclogger mechanism was also successfully demonstrated.

**Figure 7:**
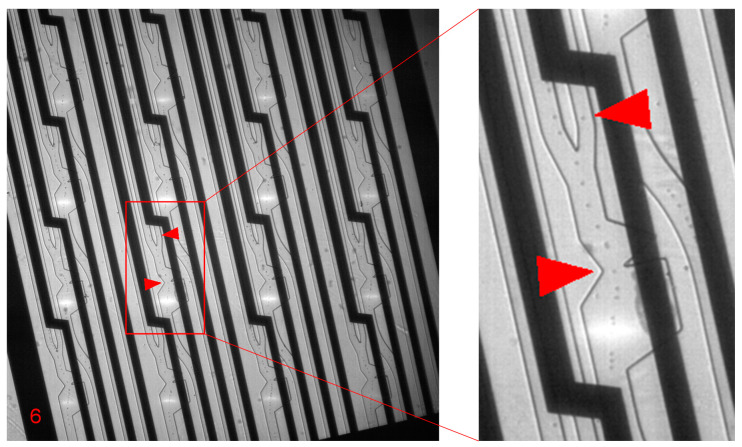
Simultaneous independent asynchronous operation of 16 individual sorters on a chip. Pictured here is a single frame from the video: see Video S1 for the full demonstration. The strobe LED and camera sample individual events from the sorters in turn. One event is pictured on individual sorter 6: full frame (**left**) and close-up (**right**). In addition to the double image of the particle at the actuation position and verification position, the actuation bubble can be seen behind the silhouette of the microresistor.

The simultaneous independent asynchronous operation of the 16 individual sorters on a chip was confirmed with beads as follows. It was possible to set the actuation timing to reliably sort in each individual sorter. Figure 7 and Video S1 show strobe images, confirming stable actuation and verification of sort events. In this example, the beads were suspended at 3 × 10^6^/mL in the input. The total throughput of the particles was 198×10^3^/s or a mean of 12.4 × 10^3^/s per channel. The positive fraction was 1.0% and the total deflection rate was 1.9 × 10^3^/s. The total flow rate was 4 mL/min or 0.25 mL/min per channel.

**Figure 8:**
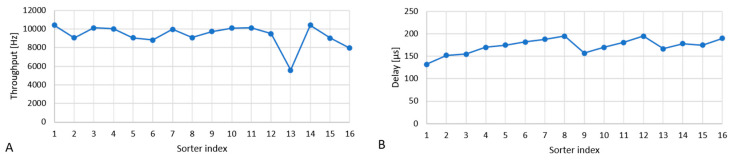
Division of particles and flow between individual sorters. (**A**): bead throughput, counted by FSC peak detection; (**B**): actuation delay timing (inversely proportional to flow velocity).

The device succeeded in dividing the particles and the flow evenly between streams. Figure 8 shows the bead throughput measured in each stream using particle counting by FSC peak detection, and the actuation delay for each stream. The latter is a measure of the stream velocity, since the actuation delay is the time period required for the particles to traverse a fixed distance between the laser focus and vortex-generating tip. While there is a variation of around 20% in the velocity, we were able to adjust the actuation delay settings so that all individual sorters functioned correctly. Except for one sorter (index 13) at the corner of the array that experienced less reliable counting due to lower intensity of the laser beam, the variation in throughput is less than around 10%.

**Figure 9:**
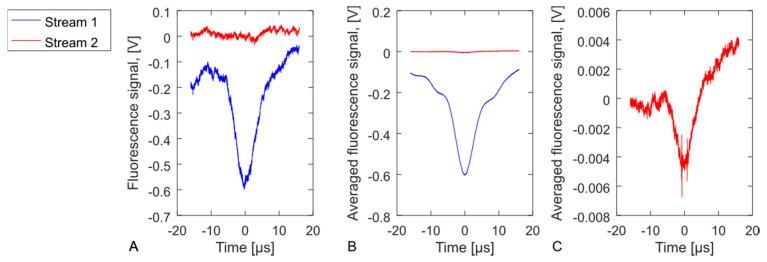
Measurement of fluorescence cytometry crosstalk between two adjacent sorters (Stream 1 and 2). We detect events (**A**) where a bead has passed through Stream 1, while no bead is detected in Stream 2. After averaging many such simultaneous traces (**B**), we compare the signal in Stream 2 with the peak of Stream 1. There is a slight peak in Stream 2 which is the effect of crosstalk, shown in full on its own axes (**C**).

The cytometry crosstalk between the individual sorters was measured by comparing FSC or fluorescence peaks simultaneously between different detector pixels using an external oscilloscope. With a sample of beads, we measured approximately 0.8% fluorescence crosstalk between neighbouring pixels, as shown in Figure 9. This appears to be due to electronic crosstalk within the multianode PMT, which agrees approximately with the specification of the PMT. The crosstalk of adjacent FSC pixels was not detectable above the noise; thus, is estimated as <0.2% including the contributions of both the optical and electronic effects.

## 4. Discussion

We succeeded in making a 16× parallel VACS chip and operating it with parallel optics and instrumentation that were set up for this purpose. The demonstration confirms the independent asynchronous operation of the individual sorters within the array, with a combined speed that is equivalent to sixteen individual sorters combined.

The effects that may have disturbed parallel sorting were either not observed, or were mild enough that we successfully compensated for them within the control parameters of the individual sorters. These included: pressure or flow transients affecting flow in neighbouring channels; the uneven distribution of particles or flow among the individual streams; difficulty in the three-dimensional alignment of the laser foci (array and plan) to the chip; and crosstalk in the cytometry channels (FSC, BSC, fluorescence) between adjacent streams.

As a raw demonstration of speed, we processed up to 60 × 10^6^ particles per run, with a run time of 5 min and an input rate of 2 × 10^5^/s, deflecting an arbitrary 1% according to FSC, BSC and fluorescence gating. This rate extrapolates to 0.7 × 10^9^/h. The channel dimensions and flow rates were identical to those in our previous paper which demonstrated sorting of peripheral blood mononuclear cells (PBMCs). If this were a single stream flow sorting device, the equivalent sort envelope rate or droplet rate would be 16 × 43 kHz or 0.7 MHz. In terms of the throughput and the equivalent droplet rate for high viability sorting, this is ten times faster than the current commercial cell sorters.

We believe it would be possible to scale-up the parallel sorter array further. Such further scaling would require the packing of inertial focuser input channels either on the chip or removal from the plane of the sorter array. Scaling would also require consideration of the optics: the collection based on a single lens can achieve the same light collection efficiency for a larger array by making the lens proportionally larger.

We succeeded in parallelizing the sorter’s optics, electronics and software sufficiently to make basic demonstrations of sorting. The quantitative data for beads are presented, since beads are easy to detect and count using our rudimentary data processing.

Much work remains to make the parallel VACS a useful and usable instrument for biological research and clinical procedures. We believe the most important work is in automation and data processing. Automated instrument control should include actuation delay timing, the alignment of the chip to the laser focus array and the detection of clogging in all individual sorters. It is also important to combine all parallel streams for simultaneous gating and presentation of data: this requires the calibration of intensities for each measurement channel across all streams.

## 5. Conclusions

Parallelisation of VACS to make an extremely high-throughput cell sorter is indeed possible, as we have shown. We set a new record for the speed of a cell sorter using conventional fluorescence and scatter cytometry, in terms of sorting lymphocyte-sized beads. The detailed sorting conditions were previously shown to be suitable for the efficient high-viability sorting of lymphocytes and small mammalian cells [28]. We believe the new result opens the possibility of upscaling cell sorting for applications requiring the processing of much greater cell numbers than currently possible using conventional cell sorters.

## Supplementary Materials

The following are available online at https://www.mdpi.com/article/10.3390/mi12040389/s1, Video S1: Parallel 16× VACS demonstration.
